# Contrast enhanced ultrasound for traumatic spinal cord injury: an overview of current and future applications

**DOI:** 10.1038/s41394-024-00644-3

**Published:** 2024-04-25

**Authors:** Brian Fabian Saway, James Courtney, Jessica Barley, Bruce Frankel, Christoph Hofstetter, Stephen Kalhorn

**Affiliations:** 1 Medical University of South Carolina, Department of Neurosurgery, Charleston, SC 29425 USA; 2 Florida State University College of Medicine, Tallahassee, FL 32303 USA; 3 Southern Illinois University School of Medicine, Department of Neurosurgery, Springfield, IL 62702 USA; 4 University of Washington, Department of Neurosurgery, Seattle, WA 98195 USA

**Keywords:** Prognostic markers, Diagnostic markers

## Abstract

**Study design:**

Systematic review.

**Objective:**

Contrast-enhanced ultrasound (CEUS) is an imaging modality that has only recently seen neurosurgical application. CEUS uses inert microbubbles to intraoperatively visualize vasculature and perfusion of the brain and spinal cord in real time. Observation and augmentation of spinal cord perfusion is vital component of the management of traumatic spinal cord injury, yet there are limited imaging modalities to evaluate spinal cord perfusion. CEUS provides an intraoperative imaging tool to evaluate spinal cord perfusion in real time. The objective of this review is to evaluate the current literature on the various applications and benefits of CEUS in traumatic spinal cord injury.

**Setting:**

South Carolina, USA.

**Methods:**

This review was written according to the PRISMA 2020 guidelines.

**Results:**

143 articles were found in our literature search, with 46 of them being unique. After excluding articles for relevance to CEUS and spinal cord injury, we were left with 10 papers. Studies in animal models have shown CEUS to be an effective non-invasive imaging modality that can detect perfusion changes of injured spinal cords in real time.

**Conclusion:**

This imaging modality can provide object perfusion data of the nidus of injury, surrounding penumbra and healthy neural tissue in a traumatized spinal cord. Investigation in its use in humans is ongoing and remains promising to be an effective diagnostic and prognostic tool for those suffering from spinal cord injury.

## Introduction

Ultrasound (US) is a non-invasive imaging modality that is safe, effective, and able to be used intraoperatively to visualize vital structures otherwise unseen by the naked eye [1]. The ability of US to be used by nearly any trained clinician has positioned it to be one of the most used imaging modalities in all fields of medicine. US has seen promising use in the field of neurosurgery, both intracranially and for spinal pathology; however, the use of contrast-enhanced ultrasound (CEUS) has only recently been explored [2]. Intravenously delivered microbubbles comprised of a lipid shell and an inert gas core serve as contrast agent. Microbubbles enhance the echo as they resonate with a specific frequency depending on microbubble diameter when reached by an ultrasound signal. Thus, contrast agent microbubbles resonate with harmonic frequencies, while adjacent tissues do not resonate which results in a superior signal-to-noise ratio of the underlying vasculature [3]. These microbubbles offer a greater resolution and image of the vascular anatomy and perfusion than conventional ultrasound techniques thus showing significant promise as an imaging modality for various forms of spinal cord pathology [4].

Traumatic Spinal cord injury (tSCI), both acute and chronic, poses a devastating threat to the health and well-being of patients, often leading to debilitating outcomes [5]. These injuries occur in multiple phases: the primary injury, and then secondary injury occurring minutes after which is due to ischemia, free radicals, and inflammatory response, all leading to changes in perfusion of the injured spinal cord [6]. While the core epicenter of injury is currently considered unsalvageable with available interventions, investigators are exploring ways to identify and treat the surrounding penumbra [7]. There is currently a lack of imaging modalities available to identify, measure, and observe this surrounding penumbra that represents potentially salvageable tissue. The quantitative and qualitative differences in the observed penumbra may require differing management strategies and may improve upon our current guidelines for treatment of tSCI.

The ability of CEUS to offer surgeons visualization of the perfusion of the spine intraoperatively, in real time, has interventional and prognostic implications in the field of tSCI. The aim of this review is to evaluate the current use and potential benefits of CEUS and its use in tSCI.

## Methods

### Search criteria

This review was written according to the PRISMA 2020 guidelines [8]. Databases searched included Pubmed, Cochrane Review, and Web of Science. Search terms used included: “CEUS AND spine”, “CEUS AND spinal cord injury”, and “CEUS AND spinal cord”. The search started in late August 2022 and continued into September of that same year, with the last database search on 9/1. Eligibility criteria was not affected by the date an article was published.

**Fig. 1:**
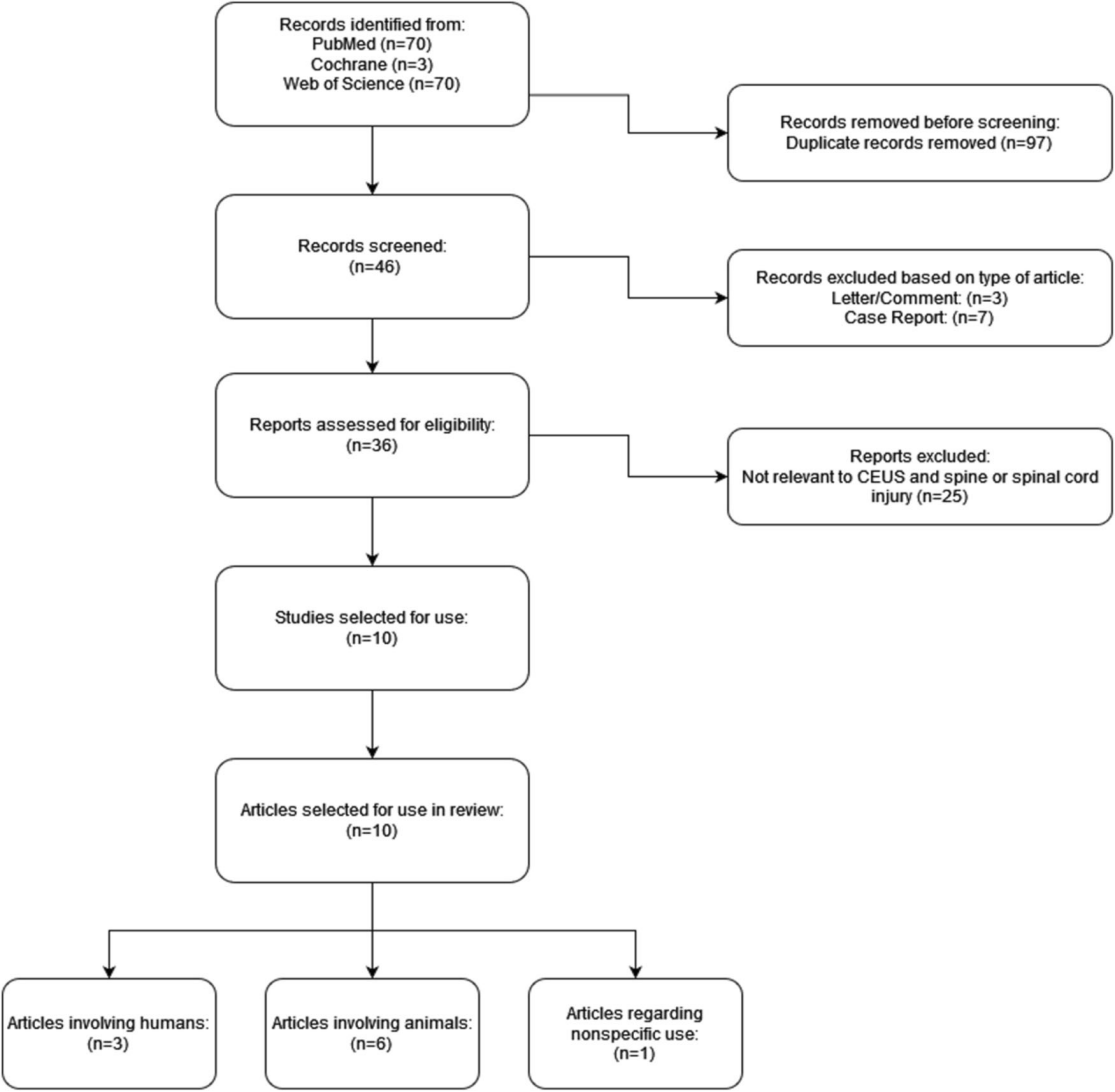
PRISMA flow diagram for review, detailing database searches, records screened, and included studies.

Records were found using the keywords listed in each database, after which duplicates were excluded. Before the screening process began, case reports, letters to the editor, and conference comments were deemed ineligible to be used in our review. There were no limits regarding the types of studies or reviews included otherwise. English as a primary language was also a requirement for selection. Figure 1 includes the PRISMA flowchart diagram, which entails our search, screening, and inclusion of reports.

### Eligibility of articles

Articles were screened for their relevance to the topic of CEUS use in spinal cord injury or spinal surgery regarding human or animal participants. Due to the novelty of the subject, animal studies were included. Papers regarding the use of CEUS in a non-specific field were also selected for their relevance to the topic. The screening process included two authors (JC and BS) in an independent review. Disagreement on article selection was then discussed which led to final selection.

## Results

143 articles were found in our literature search, with 46 of them being unique. Case reports, conference comments, and letters to the editor were excluded, leaving 36 reports available for screening. After excluding articles for relevance to CEUS and spinal cord injury, we were left with 10 papers.

## Discussion

### CEUS and spinal cord perfusion following tSCI

US has become a ubiquitous imaging modality throughout medicine, and its efficacy within the field of spinal pathology has been an area of innovation that has allowed safer surgical approaches [9]. Its real-time application, lack of ionizing radiation, relatively low cost, and dynamic modalities provides valuable intraoperative data and has led to US being found in the operating rooms of various surgical specialties [10]. Within the field of spine surgery, US can be used to visualize relevant anatomy that is outside the operator’s field of view and can be used to confirm adequate spinal cord decompression, identify neural structures, and can be used to help guide resections of the various vascular and neoplastic pathologies of the spine and spinal cord [11]. While the addition of doppler to US allows for visualization of large vascular structures, a current limitation of intraoperative ultrasound use is its abilities to identify the microvascular structures that play a large role in the perfusion of the spinal cord. This has led investigators into attempting to identify ways to acquire this valuable data intraoperatively to understand how the spinal cord parenchyma is perfused after spinal cord injury.

The importance of monitoring spinal cord perfusion after spinal cord injury has been investigated for decades and has culminated into several randomized control trials being performed to evaluate the efficacy of monitoring and augmenting spinal cord perfusion through various intradural devices [12–14]. Paralleling the treatment of traumatic brain injury which includes placement of intraparenchymal monitors, cerebrospinal fluid diversion and expansive duraplasty for the goal of targeting an optimal cerebral perfusion pressure, investigators are now promoting similar maneuvers to target an optimal spinal cord perfusion pressure (SCPP) [15]. This optimization of SCPP is performed with the goal of providing sufficient blood flow to the penumbra of salvageable neural structures surrounding the nidus of injury. In animal studies, experimental mitigation of posttraumatic, intraparenchymal swelling has shown to counteract secondary injury [16]. Despite ongoing research, guidelines for management of spinal cord injury currently includes early surgical decompression and mean arterial pressure augmentation, with no mention of monitoring or assessing SCPP. A current drawback and likely contributor to the lack of widespread use of available SCPP monitors includes their invasive characteristics and associated risk profile [17]. As current, non-invasive imaging modalities such as computed tomography (CT), magnetic resonance imaging (MRI) and digital subtraction angiography (DSA) do not provide adequate assessment of SCPP, the field of spinal cord injury is in need of a non-invasive method of evaluating SCPP following spinal cord injury.

CEUS has demonstrated itself to be a powerful diagnostic tool for visualization of perfusion of various soft tissue structures, including the spinal cord [18]. The efficacy of this modality has been realized through its leveraging of the physical properties of the contrast agent as well as the ultrasound device. The structure and size of the lipid microbubbles are less than 10 microns, smaller than the size of a red blood cell, which allows the contrast agent to flow in capillaries and arterioles, while preventing its escape out of the vessels [19]. The ultrasound probe emits a frequency that matches the harmonic frequency of the injected, intravascular microbubbles. This leads to a cyclical compression and expansion of the microbubbles, producing an echo that is then received by the piezoelectric crystals on the ultrasound probe [20]. As contrast agent microbubbles resonate with harmonic frequencies, while adjacent tissues do not resonate, this results in a superior signal-to-noise ratio of the underlying vasculature. This ultimately results in a robust signal that can be visualized by the operator. By isolating the contrast agent to the capillaries, and the resulting non-linear echogenic curve produced by the interaction of the emitted frequency and the microbubbles, one can detect in detail the perfusion of the observed tissue with minimal artifact or extraneous hyper-echogenicity [21].

The structure of the contrast agent is also designed for stability, allowing the agent to be persistent in the vasculature. The structure of a microbubble includes a lipid microsphere which encloses an inert gas such as nitrogen or sulfur hexafluoride [22]. Contrast microparticles will eventually leave the body via the lungs, breathed out as the inert gas that was inside the microbubble. These features make contrast microbubbles a safe and effective agent at improving ultrasound imaging, and investigators have now begun to explore its efficacy as a diagnostic modality to evaluate spinal cord perfusion following spinal cord injury.

A current limitation of the use of CEUS for spinal cord injury is the inability to visualize the spinal cord prior to decompression via laminectomy. The significant artifact caused by the bony, posterior elements encasing the spinal cord creates a barrier to ultrasound assessment of the underlying, injured spinal cord. An understanding of how the perfusion of spinal cord changes before and after spinal cord decompression could provide clinicians with intraoperative information that could influence the need for further decompression or duraplasty, as well as prognostic information. Nevertheless, as ultrasound technology advances and ultrasound probe size decreases while maintaining or improving on image quality, there is promise that ultrasound visualization of spinal cord via the interlaminar space or a small laminotomy can be performed and provide this essential pre-decompression perfusion information.

This review of available literature details the work of several investigators that have worked to fill the current void of perfusion imaging in the field of spinal cord injury by demonstrating the efficacy of CEUS as a diagnostic and prognostic imaging tool. A summary of the available literature discussed can be found in Table 1.

**Table 1:** Summary of available literature on CEUS and its current and future role in tSCI.

Author	Type of participant	Center(s)	Participants	Intervention	Primary Findings
Khaing [16]	Rodent	Single	6	tSCI induced and CEUS used to visualize thoracic spinal cord pre-and post- injury	CEUS was effective at visualizing perfusion and hypoperfusion before and after a tSCI
Khaing [23]	Rodent	Single	5	tSCI induced and CEUS used to visualize area of injury. Phantom system used to determine if CEUS can differentiate between moving and stationary microbubbles	CEUS was able to determine the difference between moving and stationary particles, as well as being effective at visualizing areas of hyper and hypoperfusion of the spinal cord post injury.
Han [31]	Human	Single	14	Intraoperative CEUS was used alongside preop MRI in order to create a surgical plan	Intraoperative CEUS was very effective at determining tumor size and margin. CEUS was also effective at determining tumor perfusion.
Bruce [24]	Rodent	Single	N/A	In vitro and in vivo experiments involving female rats, where tSCI was induced and perfusion was studied pre-and post-injury with CEUS at 15mHZ.	CEUS was more effective at visualizing larger vasculature with a larger velocity at 15mHz, and was able to effectively observe perfusion in real time.
Bruce [25]	Rodent	Single	N/A	Blood flow was observed post-induced tSCI of varying severities. CEUS was used to visualize perfusion of the injury sites.	CEUS was able to visualize the blood flow of the different injury sites. Higher severity of injury indicated a larger and more hypoperfused area of tissue. Moderate injury sites were more localized in their hypoperfusion, and were more symmetrical.
Della Pepa [2]	N/A	N/A	N/A	Systematic Review to understand current use of CEUS in neurological surgery	CEUS was mentioned in 67 papers from 1999 to 2019. While most studies evaluate its use intracranially, CEUS shows promise as an effective tool that can visualize spinal cord tissue in real time, in the operating room.
Ling [29]	Human	Single	12	Circular decompression of thoracic spinal cord	CEUS allowed intraoperative visualization of perfusion before and after decompression surgery.
Vetrano [30]	Human	Multi	12	CEUS was used to visualize tumors intraoperatively during removal	CEUS allowed surgeons to see perfusion and structure of tumors in real time intraoperatively.
Huang [26]	Porcine	Single	6	tSCI induced and perfusion visualized by CEUS	Spinal cord injury and subsequent hypoperfusion was visualized by CEUS. CEUS provided a view of the hypoperfusion surrounding the injury and resultant hyperperfusion adjacent to it.
Huang [27]	Rhesus monkey	Single	8	CEUS was used to visualize perfusion after induced tSCI	Hypoperfusion was found in the center of the injury site and hyperperfusion was found surrounding the adjacent area.

### Studies assessing CEUS for spinal cord injury

The use of CEUS for spinal cord injury in animal studies to date has shown significant promise for the future of spinal cord injury. Studies have been performed in rats, pigs, and most notably, rhesus monkeys. All of which have shown that CEUS can effectively visualize the perfusion changes that occur at the injury nidus and surrounding penumbra.

#### Rodent model studies

Current studies investigating the use of CEUS for spinal cord injury involving rodents have been limited to ultrafast CEUS Doppler, which is a type of CEUS developed by Khaing et al. [23]. This method involves combining CEUS with ultrafast plane-wave acquisitions, leading to visualization of high-velocity and low-velocity blood flow in the macro and microcirculation, respectively. This differentiation of the macro and microcirculation mitigates the contamination of contrast signal from other, larger vessels. All studies by Khaing involved visualizing the blood flow of the spinal cord using CEUS following induced tSCI [16, 23]. Their novel ultrafast CEUS Doppler technique enables visualization of the anterior spinal arteries, as well as the central spinal arteries. In all studies, perfusion was observed pre-spinal contusion and the gray matter was found to be higher blood flow than the white, as expected. After spinal cord injury induction, the injury site was observed using the ultrafast CEUS epidurally. Both studies showed hypoperfusion of the injury site using CEUS, and disruption of blood flow in the penumbra surrounding the contusion epicenter. Khaing et al. showed that there was a significant loss of perfusion at the site of the SCI, and that there was also a marked hypoperfusion of the penumbra in the 1st hour after injury. During the first hour after surgery the hypoperfused injury core enlarges by more than 40%. They also found that the more ventral and rostral visualized arteries deflected more rostrally following the spinal cord injury possibly caused by mass effect of the hemorrhagic necrotic injury center. The authors hypothesize that this may have an impact on the blood flow to the penumbral zone of the injury. Another study by Khaing et al. [24] used a novel transcutaneous approach to CEUS. This non-invasive method was proven to be feasible by showing similar results of hypoperfusion by transcutaneous approach, although there was some signal loss and attenuation compared to the previously studied epidural methods of CEUS. The confirmation of this transcutaneous approach has significant implications as this would expand the utility of CEUS for spinal cord injury outside of the operating room. A transcutaneous capability of CEUS to monitor SCPP of a decompressed spine following skin closure would allow for objective data to guide the medical management provided in the postoperative period. Bruce et al. published studies regarding the use of high frequency CEUS at 15 MHz [25, 26]. These studies involving rodents showed a significant difference in the perfusion depending on the severity of injury. Severe injuries showed a more rostral-caudal projection of damage as well as a larger hematoma size. CEUS at this higher frequency also demonstrated differences in the vasculature at and around the spinal cord depending on if a contusion was moderate or severe. In rodents with severe injuries, there was almost twice as much hypoperfusion in the microvasculature compared to moderate injury. Moderate injuries showed a more symmetric area of injury as well. Importantly, the degree of lesional hypoperfusion correlates with the injury severity [26]. These studies in rodents show that by using CEUS it is possible to get real time, high-quality images of the perfusion of the spinal cord and injury site.

#### Porcine model studies

Using a porcine model, Huang et al. [27] evaluated tSCI with CEUS in 6 female, juvenile pigs. Similar to other animal models, they found that the healthy porcine spinal cord was hypoechoic and homogenous, whereas the dura and pia mater were hyperechoic on conventional, non-contrasted ultrasound. After inflicting traumatic injury to the spinal cord, they visualized the injured spinal cord with conventional US and found that there was significant hypo-echogenicity in the injured area of the cord. As the observation period went on, adjacent intramedullary blood vessels also became dilated. The authors also used Color Flow Doppler Imaging (CDFI) to observe that the injured intramedullary vessels were dilating. Using CEUS, they found that the epicenter of the injury was hypoperfused, and that the adjacent penumbra hyperperfused, with significantly different peak intensities found in these areas. Like other animal studies discussed, histological analysis was performed to determine the extent of spinal cord injury, confirming the presence and distribution of spinal cord injury demonstrated on CEUS. In this study, the authors were able to successfully visualize perfusion and hypoperfusion of spinal cord injury in a porcine model.

#### Rhesus monkey model studies

CEUS to evaluate spinal cord perfusion following spinal cord injury has also been studied in rhesus monkeys. One study used 8 rhesus monkeys of both genders, all aged at 4 years old [28]. Perfusion of the white and gray matter was visualized using conventional ultrasound, where there were expected hypoechoic and homogeneous patterns in the healthy spinal cord. The authors used Doppler flow imaging to observe the intramedullary vessels of the spinal cord in the sagittal plane. Subsequent injury to the spinal cord showed a gradually increasing hyperechoic center of damage on conventional ultrasound. CEUS demonstrated that the center of the injury lacked perfusion, and on conventional ultrasound had a heterogeneous echogenicity, compared to the initial homogeneity seen pre-spinal cord injury. While the authors found hypo-perfusion at the site of injury, there were areas of hyperperfusion revealed on CEUS in the adjacent areas around the injury, rostrally and caudally. The heterogenous perfusion at the injury penumbra was an intriguing discovery and may contribute to the variable improvement in function following spinal cord injury and the variable efficacy of MAP augmentation seen on humans that suffer from spinal cord injury [29]. Histological analysis in this study demonstrated a cavity lesion with glial scarring around it, as well as demyelination in the adjacent areas to the injury, paralleling the findings seen on CEUS. The use of CEUS in this study provided the authors with a clear picture of the real-time perfusion changes following a primate SCI, which shows its practicality for use in human SCI.

#### Human studies

While there is currently only one ongoing clinical trial investigating the role of CEUS following tSCI (NCT04056988), no study to date has been published exploring this topic of spinal cord injury and CEUS in humans. Despite this, there have been several studies using this imaging modalities for various other spinal cord pathologies in humans. Ling et al. [30] performed a clinical study of 12 patients undergoing surgery for decompression of chronic compressive thoracic vertebrae using CEUS. The authors were looking at time-intensity curves, enhanced intensity of the image, and rise time. Ling measured differences between normal spinal cord, damaged spinal cord, and the spinal cord after undergoing ventral decompression surgery. Using CEUS after decompression, the authors appreciated the difference in the peak intensity of the image and the background. There was a statistically significant difference in the enhanced intensity between a decompressed spine and the normal spinal cord (*p* < 0.04). There were no other significant findings from the study except for the difference in cord perfusion (measured as the slope of the time-intensity curve) with a *p* value of 0.02. Although this study did not pertain to tSCI decompression, the use of CEUS and its value is demonstrably shown.

A retrospective study by Vetrano et al. [31] looked at 12 patients undergoing intramedullary spinal tumor resection. This study used CEUS to solely look at the vascularization pattern and flow characteristics. Although there were various types of intramedullary tumors present in the patient sample, CEUS helped to clearly delineate tumor boundaries except in the case of an anaplastic astrocytoma and a GBM. The authors found that CEUS easily allowed the surgeon to visualize the tumor border with its homogeneous characteristics compared to the background. There was another prospective study regarding the use of CEUS in the resection of intramedullary spinal cord tumors, authored by Han et al. [32]. Han took 14 patients with various tumors and used CEUS during their resection. Using CEUS, the surgeons saw clear boundaries of each tumor and were able to resect the masses successfully. The authors specifically saw that large tumors, usually with boundaries which are hard to define, were easily delineated by CEUS. While this does not directly pertain to tSCI, it provides evidence that this imaging modality can be safely administered in humans and can provide valuable spinal cord perfusion data. Altogether, these studies demonstrate the promising ability of CEUS to be used as an intraoperative imaging modality in humans to understand the perfusion to an injured spinal cord following trauma.

### Future directions

It is clear through the various animal and human studies currently published that CEUS can effectively detect blood flow changes the spinal cord. With this foundation established, future directions must be focused on evaluating the efficacy of CEUS to identify these perfusion changes in tSCI occurring in humans. One could envision that these perfusion changes identified by CEUS can be used in synergy with intraoperative neuromonitoring to confirm signal changes that occur during spinal cord decompression. Furthermore, CEUS may help us understand the etiology, pathophysiology, and preventative measures of elusive pathologies such as reperfusion injuries (i.e., “white cord syndrome”) and C5 palsy that occur after surgical decompression for chronic spinal cord injury from degenerative spine conditions leading to myeloradiculopathy. Interestingly, all studies involving porcine and rhesus subjects, included juvenile subjects [27, 28]. It is not uncommon for human patients suffering from tSCI to have underlying degenerative spine disease and chronic spinal compression. The perfusion changes that occur after decompression of patients that have tSCI and superimposed chronic spinal cord injury may be different than what is seen in a healthy spinal cord and could help guide prognostic discussions. Further research assessing spinal cord perfusion with CEUS in patients with chronic spinal cord compression is needed to help answer these important questions.

Given the mounting evidence of CEUS being an effective tool for evaluating spinal cord perfusion, the clinical application and implications are vast. The degree of perfusion changes identified could guide the degree and timeframe for use of blood pressure augmentation, CSF diversion with placement of a lumbar drain, and the need for expansive duraplasty. The work by Khaing et al. [24] providing evidence of the efficacy of transcutaneous CEUS to identify spinal cord perfusion changes in rodents shows the future applicability of bedside applications of this modality following surgical decompression. In this future application, serial transcutaneous CEUS imaging could feasibly be performed in the ensuing days following spinal cord injury to guide medical management and obviate the need for intradural spinal cord perfusion measuring devices that are currently being explored.

Moreover, this imaging modality shows potential to be used as a prognostic tool where perfusion changes identified in the penumbra can help predict the degree of possible recovery. It is possible that both the size and the relative extent of the penumbra of hypo- and hyperperfused spinal cord as compared to the injury nidus can be used as an objective marker for degree of permanent spinal cord injury as well as potential for clinical recovery.

## Conclusion

CEUS is a non-invasive imaging modality that has been shown in animal models to effectively detect perfusion changes in traumatically injured spinal cords. This imaging modality is being actively investigated in humans and shows promise to be an effective diagnostic and prognostic tool for patients suffering from spinal cord injury.

## Data Availability

Data sharing is not applicable to this article as no datasets were generated or analyzed during the current study.
